# Mothercraft and Child Welfare

**Published:** 1917-06-09

**Authors:** 


### MOTHERCRAFT AND CHILD WELFARE.

### Maternity as State Service.

The women students of University College were fortunate in having Lady Barrett to lecture to them on May 25 on Infant Welfare. The growth of the present condition of public opinion, the action taken, and the pressing claims of the future were summarised by her in the way most useful to young women in this critical time. The root of the child's welfare was now seen to be the mother's condition. The lecturer's own experiment in giving, at a pre-war cost of 2d., a meal of meat and vegetables with pudding and fruit to expectant mothers, who too often lived on tea and bread, had often given the baby its right nourishment. Medical inspection in schools had revealed a startling amount of ill-health, and, as Sir George Newman said in his great report, it was too late to make these children into the men and women they ought to be. Yet we saw how the grown lads of our New Army developed in right conditions of food, fresh air, sleep, exercise. The influence of body on brain must not be forgotten, many children of mothers constantly themselves suffering from "fatigue poison" are only half alive. The Insurance Act revealed the ill-health of women to such an extent that their supposed malingering was inquired into. Work such as that of Alderman Broadbent showed the needlessness of much infant mortality and led to the Notification of Births Act. Again, the "toddlers" have been found to be a problem and to deteriorate and sicken when a new baby is taking up the mother's time and attention and the stage of school care is not reached. Pure milk, clean and sufficient water, decent housing, sufficient money to meet the expenses of a birth, proper care and rest for herself, these are the crying needs of the poor mother. Voluntary centres of work could never be sufficient; there must be municipal schemes, but the work of volunteers should keep them human. As to the cost, a ½d. rate would provide the centres; but all the pre-natal and special care and relief needed could be supplied by a 2d. rate, or with 50 per cent. from the Treasury a 1d. rate would suffice. Only, Councillors must be aroused, and women must be on all Councils, every one of which should have a Maternity Sub-Committee. If to the present 150,000 yearly deaths of infants, and probably equal number of pre-natal losses, are added the national loss of the maimed, the inefficient, and the population of asylums, then the lives sacrificed on the present battle-fields are not more tragic nor more wasteful. To save them must be everybody's business—"let it be theirs in particular," Lady Barrett concluded. It may be worth while now and again to remember that only a few years ago the minds of many thinkers were haunted by a contrasting sense of calamity. Not the deaths but the lives of the children of poor homes were the subject of anxiety. Processions of unemployed filled our streets each winter. Deplorable lads hung about idle at corners, men over forty feared being dismissed to make room for others. Girls crowded into unskilled work, placards were frequently hung outside factories and workrooms "No more hands required," yet land went out of cultivation, and great spaces of the Empire were unpeopled. Well, the wheel has come full circle!

And now we have the formulated demand for endowment of motherhood put forward as a matter of practical politics. It is only the logical outcome of the conclusions arrived at by all the inquirers and students. The Memorandum* prepared by the Women's Co-operative Guild involves schemes as wide, as thorough, and we may add as costly, as those for education which have been formulated by the Labour Party and other bodies. If the postulate that maternity is a direct service to the State be accepted, it follows naturally that "every" mother is as much entitled to her wages as "every" member of Parliament to his £400 a year. The Co-operative Guild frankly, though briefly, include the unmarried mother. They ask that a State Maternity Department should at once be formed, devolving powers upon Maternity Committees of Councils. Fifty such committees, we note, already exist. Working women should be everywhere appointed on the administration, since ladies who have sat on charitable committees may be out of touch with the point of view of independent working women. This is a point on which the Guild lay special stress, a fact that is very significant. Midwives should be municipal officials at a minimum salary of £150, and in addition home helps should also be municipally provided to manage the house during the mother's disablement. Maternity hospitals, provided with ambulances, pre-natal consultations, mothers' dinners, provision for difficult cases, convalescent homes, all naturally form part of the scheme, with special grants for the promotion of research work. But, in addition to all these maternity services, which "women should feel as free to use as they do to use the Council schools"—we note that compulsion is not yet suggested—there should be a maternity allowance of not less than 10s. weekly for six weeks to all women whose husbands' wages are below £160, the old limit for income tax. This would, we may gladly notice, bring in the wives of many poor clergy.

Will this or anything like it be done? Those who have read that most piteous volume published by the Guild, entitled "Maternity, Letters from Working Women," cannot fail to have their hearts touched to feel and their brains roused to think. Workers among the poor can testify to the incredulous amusement as at hearing some fascinating fairy-tale with which an audience of working women will commonly receive the suggestion of such State action. No doubt any serious attempt at legislation will meet with strenuous opposition. Such opposition will surely fasten upon such points especially as the vast cost involved, and the premium that might be set upon immorality by the inclusion of the unmarried mother. We may notice as a sign of the changed feeling in this respect Dr. Hope's remark in the Carnegie Trust Report that the claims of the illegitimate child are very insistent. Prejudice, which led many maternity hospitals to refuse treatment, is fast disappearing. Opposition, however, means discussion, and discussion means a remoulding of public opinion, and this cannot begin too soon. We have learnt to be ashamed of our losses in children dead and children disabled, we shall soon be ashamed to allow mothers to have laid upon them a burden altogether too heavy to bear.

* *Memorandum on the National Care of Maternity.* Price 3d. Women's Co-operative Guild, 28 Church Row, Hampstead, N.W.

